# Survivin Promoter Polymorphism (-31 C/G): A Genetic Risk Factor for Oral Cancer

**DOI:** 10.31557/APJCP.2019.20.4.1289

**Published:** 2019

**Authors:** Rehana Faryal Mehdi, Fouzia Sheikh, Rizma Khan, Bina Fawad, Ahteshaam Ul Haq

**Affiliations:** 1 *Department of Pathology, Ziauddin University Clifton campus,*; 2 *Department of Molecular Genetic, Ziauddin University North Nazimabad Campus,*; 3 *Department of Community Medicine, Ziauddin University Clifton Campus, Karachi, Pakistan. *

**Keywords:** Oral squamous cell carcinoma, Survivin, 31G/C polymorphism

## Abstract

**Background:**

The polymorphism of survivin gene at its promoter region is one of the risk factors for OSCC . This polymorphism involves substitution of G for C (9904341), and it is present at the cell cycle dependent elements and cell cycle homology region repressor binding motif of promoter. This study aimed to find the association between survivin -31C/G polymorphism and prevalence of OSCC in a subset of Pakistani population.

**Methodology:**

This case-control study was conducted on 47 cases with and 101 healthy individuals with no family history of cancer. We used polymerase chain reaction and restriction fragment length polymorphism (PCR-RFLP) protocols.

**Results:**

The most common site of oral cancer in our research was the buccal mucosa followed by tongue and the least one was the labial mucosa. The histological tumor type of all 47 cases was squamous cell type. In our research, stage II had the highest prevalence, accounting for 34% of patients, while the prevalence of stage I was 31% in the case group. The prevalence of stage III and IV was 25% and 8%, respectively. The numbers of moderately and poorly differentiated tumors were equal. We found a significant association between the CC genotype of survivin and OSCC prevalence (OR was 9.395 at 95% CI: 1.0202-86.5251, p-value= 0.04). The GG genotype also showed significant P value (OR: 0.4709 with 95% CI: 0.2323- 0.9546 at a P VALUE of 0.0367). while no significant P value was noted for CG genotype (OR: 1.4317 with 95% CI: 0.7513 -2.8658, p- value= 0.31).

**Conclusion:**

Survivin -31G/C polymorphism was strongly associated with OSCC prevalence. The C allele was more common in case group as compared to healthy individuals living in Pakistan.

## Introduction

Cancer remains the deadliest disease worldwide with increasing mortality. The burden of this disease remains evenly distributed in both the developed as well as the less developed countries, although the type of cancer may vary in both. Lung cancer is more common in the developed countries as compared to stomach and oral cancer in the less developed countries. The oral cancer ranks the eighth most common cancer in males in the developing countries (Torre et al., 2015). The South Asian countries like Pakistan, India, Srilanka, and Bangladesh has considerably higher prevalence of oral squamous cell carcinoma (OSCC). This can be attributed to similar cultural practices and habits like smoking, alcohol abuse, and consumption of chewable tobacco products (Moore et al., 2010; Rao et al., 2013). In Pakistan alone, the prevalence of OSCC is different from 7.0 – 9.9% (Ahmad et al., 2007). An increasing trend is seen in cheek and tongue cancer in both sexes, with lip cancer decreasing in Asian countries. The mean age of oral cancer presentation is between 51 to 70 years, but cases with OSCC are also being diagnosed nowadays in patients less than 40 years . Men are affected more with OSCC as compared to women in most countries. For instance, males are affected 1.5 times more as compared to females in Pakistan; however, the prevalence of OSCC is higher among women living in Thailand (Rao et al., 2013). 

The habitual risk factors like smoking, alcohol abuse, and consumption of chewable variety of tobacco contribute immensely to the development of oral cancer. Chewing of betel quid, areca nut, and their substitutes like gutka, naswar, and Manipuri, as a habit, is mostly seen in countries like Pakistan, India, and Bangladesh due to similar cultural backgrounds and practices (Khan et al., 2012; Markopoulos, 2012). Viruses like Epstein bar virus (EBV) and human papilloma virus (HPV) have also strong association with the development of OSCC. OSCCis more common in people who have the habit of eating betelquid, gutka, and manipuri (Acharya et al., 2015; Sad et al., 2014). Besides these risk factors, the genome of a human also shows variation, which is called single nucleotide polymorphism (SNP). SNPs are low penetrance genes that are seen in >1% of ethnic population. Resistance to apoptosis, cellular proliferation, and angiogenesis can be caused by deregulation of functioning genomic SNPs.

Many studies have shown a strong association between SNP and the risk of breast, lung, and oral cancers (Goode et al., 2002; Choudhury et al., 2015).

One such SNP is of survivin. Survivin is a protein that belongs to inhibitor of apoptosis protein family (IAP). It is the smallest member of this family containing 142 amino acids, and it functions to inhibit apoptosis. Survivin is found minimally in adult tissues and also plays a major role as cell cycle modulator. It is highly expressed in G2/M phase. Large number of malignancies exhibit higher expression of survivin and it is associated with the aggressive forms of the disease. The gene of surviving spans 14.7kb in the telomeric region of chromosome 17q25. It produces 5 different proteins through alternate splicing of pre mRNA (Jaiswal et a., 2013; Mobahat et al., 2014).

Many of SNPs have been noted in the promoter region of this gene. Among the popular ones is -31 located in the promoter region which is present in a number of malignancies. This polymorphism involves substitution of G for C (9904341), and it is present at the cell cycle dependent elements and cell cycle homology region repressor binding motif of promoter. Its expression correlates with translation and transcription level of survivin (Multani and Saranath, 2016). A study done in Iran showed that GC and CC genotypes were significantly higher in stage III and IV of OSCC as compared to stage I and II, but it failed to find out any association between survivin -31 SNP and grading of OSCC (Mostaan et al., 2013). Given the fact that survivin is a prognostic marker in many cancers, it seems that there are some therapeutic implications which need to be worked upon in relation to conventional chemotherapy (Kim et al., 2010) .Therefore, we compared the prevalence of -31 SNP of survivin in oral cancer patients and normal healthy adults with no prior history or family history of cancer.

## Materials and Methods

This case control study was done at Ziauddin University Karachi. The project was carried out from April 2018 to June 2018. The present research was approved by Ethics Committee of Ziauddin University. 

*Sampling Criteria*

The sample size was calculated by OPEN EPI version 3. 1 case : 2 control ratio was used. Sampling was done using consecutive technique . The number of cases were 47 while controls were 101. Patients were selected among those who referred to OPD of Dr. Ziauddin dental hospital and Dr. Ziauddin Oncology clinics. Informed consent was obtained from all the participants. Participants in the control group had no family history of cancer. Data on participants’ demographic characteristics and information about exposing to carcinogens were collected by a questionnaire. Tumor differentiation was examined by the pathologist and rated according to AJCC classification. *Sample Collection*

First, 3cc blood was collected in EDTA tubes for both control and case groups. Samples were centrifuged for 10 minutes at 4^o^C at 4,000 rpm.

*DNA extraction*

DNA was extracted using GeneJET Genomic DNA putrification kit of Thermoscientific and according to manufacturer’s protocol. Extracted DNA was stored at -20^o^C.

*PCR amplification*

The reaction mixture contained 25µl of PCR Mastermix 2x, 2 µl of diluted forward primer, 2 µl of diluted reverse primer, 11 µl of nuclease free water, and 10 µl of extracted DNA. Reaction was performed on a conventional PCR under the following cycling conditions: initial denaturation at 95^o^C for 10 minutes, followed by 40 cycles of denaturation at 95^o^C for 30 seconds, annealing at 52^o^C for 45 seconds, extension at 72^o^C for 30 seconds, and a final extension at 72^o^C for 7 minutes.

**Table 1:** shows the Demographic and Clinicopathological Features of the Cases and Controls Included in the Study

Variables	Cases		Controls	
	N	%	N	%
Gender				
Female	9	19	55	54
Male	38	81	45	45
Ethnicity				
Balochi	0	0	1	1
Others	3	6	12	12
Pukhtoon	3	6	9	9
Punjabi	2	4	20	20
Sindhi	7	15	12	12
Urdu Speaking	30	64	43	43
(Blank)	2	4	4	4
Tobacco Use				
Naswar	7	15	1	1
Gutka	26	55	0	0
Betel Nuts	5	11	0	0
Pan	14	30	1	1
Smoking	5	11	5	5
Location Of Oscc				
Buccal	39	83	0	0
Lips	1	2	0	0
Tongue	6	13	0	0
Not Available	1	2	101	100
Genotype				
Gg	18	38	58	57
Cg	25	53	42	42
Cc	4	9	1	1

**Figure 1:**
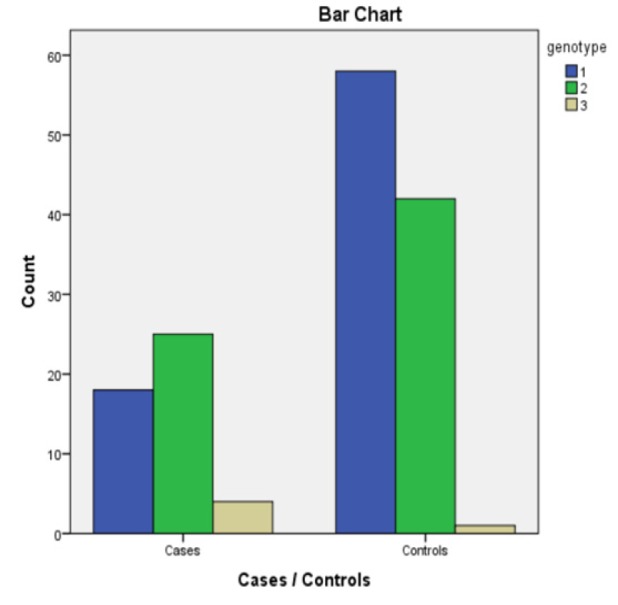
A Bar Chart Showing Frequency of Genotypes in Cases and Controls. GG=1, CG=2, CC=3.

**Figure 2:**
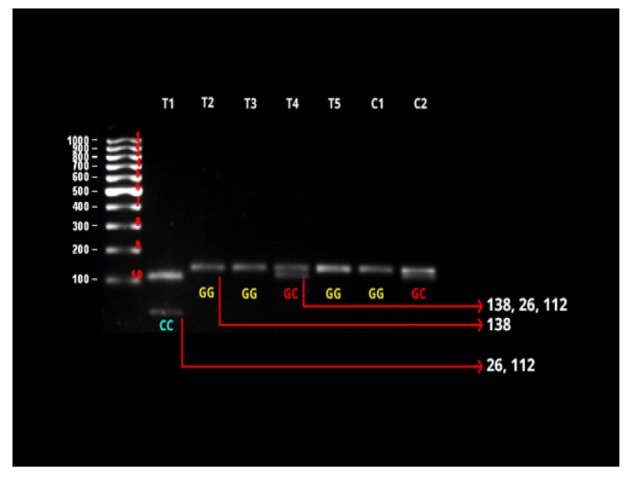
RFLP Analysis of Survivin -31G/C Gene Polymorphism. T=tumor/cases, C=controls. Lane left: 100bp DNA ladder. Lanes (2, 3, 5 and 6): GG genotype Lanes (4 and 7): GC genotype. Lane (1) CC genotype

**Table 2:** Shows the Association of the Genotypes in Cases and Controls. The results show that the GG genotype is protective to OSCC (OR=0.47, CI=0.23-0.95) while the CC genotype is at risk of OSCC

Genotype		Cases	Controls	Total	Odd’s Ratio	95% CI	P Value
Gg	Present	18	58	76	0.4709	0.2323-0.9546	0.0367
	Absent	29	44	73			
Gc	Present	24	43	67	1.43	0.7513- 2.865	0.31
	Absent	23	59	82			
Cc	Present	4	1	5	9.3	1.02-86.52	0.04
	Absent	43	101	144			

*Enzyme digestion*

Thermo Scientific MspI (HpaII) restriction enzyme was used for enzyme digestion. The reaction mixture contained 2ul of MspI enzyme, 13.5ul of distilled water, 2ul of buffer, and 12.5 microlitre of PCR product, and then it was incubated at 37^o^C for 6 hours. The digested products yielded an uncut fragment of GG, 26, and 112 bp for CC and 138, 26, 112bp of CG genotype . They were visualized on 2% agrose gel.

*Statistical Analysis*

Data were analyzed using SPSS (version 20). All the quantitative variables were expressed as mean and standard deviation, while quantitative variables were presented as frequency and percentages. The association between survivin -31 G/C polymorphism and OSCC was evaluated by running chi square test. Odd’s ratio (OR) was calculated by logistic regression at 95% confidence interval (CI). P-value less than 0.05 was taken significant.

## Results

*Clinico-pathological characteristics*

The frequency of male and females with cancer were 38 and 9, respectively. While the control group comprised of 45 males and 55 females. The mean age of controls was 43 years old and that of cases was 49 years old.

The most common site of oral cancer in our patients was the buccal mucosa followed by tongue and the least one was the labial mucosa. The histological tumor type of all 47 cases was squamous cell type. In our research, stage II was the most common accounting for 34% of the patients, while 31% were in stage I included . The frequency of stage III and IV was 25% and 8%, respectively. There was an equal amount of moderately and poorly differentiated tumor. 

*Association of OSCC with survivin genotype*

The GG genotype was present in 18 cases and 58 controls, the OR was 0.47 considering 95% CI= 0.2323- 0.9546. P VALUE was 0.0367 which is statistically significant. The CG genotype was found in 24 cases and 43 controls, OR was 1.43 considering 95% CI= 0.7513 -2.8658 (p-value=0.31) . The CC genotype was found in 4 patients in the case group and 1 patient in the control group, the calculated OR was 9.4 at 95% CI= 1.0202-86.5251 (p-value=0.04).

## Discussion

Despite myriad researche and latest treatment modalities on oral cancer worldwide, its overall 5 year survival rate has not exceeded from 45 or 50% since past 20 years. The mean age of OSCC onset is between 31 to 70 years in Asia with higher prevalence among males (Rao et al., 2013; Alamgir et al., 2013). Similarly, the mean age of our patients in the case group was 49 years old, of whom 81% were males. Our study showed that buccal mucosa hosted a large number of oral cancer cases as compared to other sites such as tongue and lip. Other studies done in Karachi and India also showed that buccal mucosa was a predominant site of OSCC as compared to tongue cancers which are much common in US population (Mirza et al., 2016; Sarode et al., 2018). The large number of cancers appearing in buccal mucosa is mainly due to consumption of carcinogenic chewable tobacco products that are kept in the buccal pouch area for longer duration (More and D’Cruz, 2013; Tanaka and Ishigamori, 2011).

Cancer, as a multietiological disease, is caused by many causative agents, including the human genome. The human genome houses SNP which predicts the possibility of encountering cancer at any time in life . Our research found an association between SNP of survivin and OSCC (Tanaka and Ishigamori, 2011; Multania and Saranath, 2016; Shojaei et al., 2018). Various studies showed that survivin -31G/C polymorphism was positively correlated with the development and aggressiveness of urothelial, colorectal, gastric, lung, breast, and oral carcinomas (do Nascimento Borges et al., 2011; Jang et al., 2008). This polymorphism is present in the promoter area of survivin gene which activates the transcription of survivin protein and hence is an attractive target for gene therapy regarding cancer prevention of (Xu et al., 2004; Bao et al., 2002). A met analysis was done on Pakistani population in 2012 and showed that survivin C allele had 1.27 fold potential for causing cancer as compared to G allele in Asian population. In addition, the aforementioned study also revealed that the patients who carried the C allele were at a probable risk of oral cancer (Srivastava et al., 2012). In our study, CG genotype was seen more commonly in oral cancer patients (OR=1.4317 at 95% CI= 0.7513 -2.8658, p-value= 0.31) as compared to GG (OR= 0.4709 at 95% CI= 0.2323- 0.9546, p-value= 0.0367) and CC (OR=9.395 at 95% CI= 1.0202-86.5251, p-value= 0.04) genotype. A similar study in Iran also reported the same results, further supporting our findings (Mostaan et al., 2013). Survivin C allele is also reported as a risk factor in colorectal and nasopharyngeal carcinomas (Gazouli et al., 2009; Ma et al., 2011). On the contrary, studies done in Taiwan and Serbia showed that the G allele was the one which carried the risk of oral cancer (Weng et al., 2012; Kostić et al., 2013). This polymorphism in the promoter area of survivin is correlated with the increase of survivin expression both at mRNA level and protein level. An exaggerated amount of produced survivin thus will hinder apoptosis and facilitate cancer formation and aggressiveness (Xu et al., 2004; Wang et al., 2012; Zhu et al., 2013). Therefore, survivin rs9904341 is considered as a threat specially among Asians (Qin et al., 2014; Zhu et al., 2013). At-risk Individuals should be screened for this polymorphism in order to come up with better prognosis and treatment regime.

In conclusion, the present study revealed that the C allele of survivin at position number -31 was significantly associated with the risk of oral cancer in a subset of Pakistani population. Our findings also highlighted the probable role of C allele of survivin in the pathogenesis of OSCC; therefore, it can be useful for formulating therapeutic strategies and diagnostic implication keeping in mind the Ethnicity of Asians and the allele which is commonly found in them.

## Conflict of interest

No conflict of interest in terms of research, authorship.

## References

[B1] Acharya S, Ekalaksananan T, Vatanasapt P (2015). Association of Epstein-Barr virus infection with oral squamous cell carcinoma in a case–control study. J Oral Pathol Med.

[B2] Ahmad Z, Azad NS, Yaqoob N (2007). Frequency of primary solid malignant neoplasms in both sexes, as seen in our practice. J Ayub Med Coll.

[B3] Alamgir M, Jamal Q, Jafarey N, Mirza T (2013). Clinicopathological parameters of 50 oral squamous cell carcinoma cases in Karachi. Pak J Med Sci.

[B4] Bao R, Connolly DC, Murphy M (2002). Activation of cancer-specific gene expression by the survivin promoter. J Natl Cancer Inst.

[B5] Choudhury JH, Singh SA, Kundu S (2015). Tobacco carcinogen-metabolizing genes CYP1A1, GSTM1, and GSTT1 polymorphisms and their interaction with tobacco exposure influence the risk of head and neck cancer in Northeast Indian population. Tumor Biol.

[B6] do Nascimento Borges B, Burbano RR, Harada ML (2011). Survivin-31C/G polymorphism and gastric cancer risk in a Brazilian population. Clin Exp Med.

[B7] Gazouli M, Tzanakis N, Rallis G (2009). Survivin-31G/C promoter polymorphism and sporadic colorectal cancer. Int J Colorectal Dis.

[B8] Goode EL, Ulrich CM, Potter JD (2002). Polymorphisms in DNA repair genes and associations with cancer risk. Cancer Epidemiol Prev Biomarkers.

[B9] Jaiswal PK, Goel A, Mittal R (2015). Survivin: A molecular biomarker in cancer. Indian J Med Res.

[B10] Jang JS, Kim KM, Kang KH (2008). Polymorphisms in the survivin gene and the risk of lung cancer. Lung Cancer.

[B11] Khan MA, Saleem S, Shahid SM (2012). Prevalence of oral squamous cell carcinoma (OSCC) in relation to different chewing habits in Karachi, Pakistan. Pak J Biochem Mol Biol.

[B12] Kim YH, Kim SM, Kim YK (2010). Evaluation of survivin as a prognostic marker in oral squamous cell carcinoma. J Oral Pathol Med.

[B13] Kostić M, Nikolić N, Ilić B (2013). Analysis of polymorphism in the survivin gene promoter as a potential risk factor for head and neck cancers development. Srp Ark Celok Lek.

[B14] Ma F, Zhang H, Zhai Y (2011). Functional polymorphism-31C/G in the promoter of BIRC5 gene and risk of nasopharyngeal carcinoma among chinese. PLoS One.

[B15] Markopoulos AK (2012). Current aspects on oral squamous cell carcinoma. Open Dent J.

[B16] Mirza D, Raza G, Basit A (2016). Oral squamous cell carcinoma (Oscc) in Karachi city-A retrospective study. Pak Oral Dent J.

[B17] Mobahat M, Narendran A, Riabowol K (2014). Survivin as a preferential target for cancer therapy. Int J Mol Sci.

[B18] Moore MA, Ariyaratne Y, Badar F (2010). Cancer epidemiology in South Asia-past, present and future. Asian Pac J Cancer Prev.

[B19] More Y, D’Cruz AK (2013). Oral cancer: Review of current management strategies. Natl Med J India.

[B20] Mostaan LV, Tabari A, Amiri P (2013). Survivin gene polymorphism association with tongue squamous cell carcinoma. Genet Test Mol Biomarkers.

[B21] Multani S, Pradhan S, Saranath D (2016). Gene polymorphisms and oral cancer risk in tobacco habitués. Tumor Biol.

[B22] Multani S, Saranath D (2016). Genotypic distribution of single nucleotide polymorphisms in oral cancer: global scene. Tumor Biol.

[B23] Multania S, Saranath D (2016). Single nucleotide polymorphisms and risk of oral cancer: Indian Case-Control Study. J Clin Cell Immunol.

[B24] Qin Q, Zhang C, Zhu H (2014). Association between survivin-31G> C polymorphism and cancer risk: meta-analysis of 29 studies. J Cancer Res Clin Oncol.

[B25] Rao SVK, Mejia G, Roberts-Thomson K, Logan R (2013). Epidemiology of oral cancer in Asia in the past decade-an update (2000-2012). Asian Pac J Cancer Prev.

[B26] Sand L, Jalouli J (2014). Viruses and oral cancer Is there a link?. Microb Infect.

[B27] Sarode SC, Sarode GS, Patil S (2018). Decoding oral cancer conundrums from ‘bad luck’point of view. Future Med.

[B28] Shojaei F, Yazdani-Nafchi F, Banitalebi-Dehkordi M, Chehelgerdi M, Khorramian-Ghahfarokhi M (2018). Trace of survivin in cancer. Eur J Cancer Prev.

[B29] Smitha T, Mohan C, Hemavathy S (2017). Clinicopathological features of oral squamous cell carcinoma: A hospital-based retrospective study. J Dr NTR Univ Health Sci.

[B30] Srivastava K, Srivastava A, Mittal B (2012). Survivin promoter− 31G/C (rs9904341) polymorphism and cancer susceptibility: a meta-analysis. Mol Biol Rep.

[B31] Tanaka T, Ishigamori R (2011). Understanding carcinogenesis for fighting oral cancer. J Oncol.

[B32] Torre LA, Bray F, Siegel RL (2015). Global cancer statistics, 2012. CA Cancer J Clin.

[B33] Wang X, Huang L, Xu Y (2012). Association between survivin− 31G> C promoter polymorphism and cancer risk: a meta-analysis. Eur J Hum Genet.

[B34] Wang Y-H, Chiou H-Y, Lin C-T (2009). Association between survivin gene promoter− 31 C/G polymorphism and urothelial carcinoma risk in Taiwanese population. Urology.

[B35] Weng C, Hsieh Y, Chen M (2012). Survivin SNP-carcinogen interactions in oral cancer. J Dent Res.

[B36] Xu Y, Fang F, Ludewig G, Iones G, Jones D (2004). A mutation found in the promoter region of the human survivin gene is correlated to overexpression of survivin in cancer cells. DNA Cell Biol.

[B37] Zhu Y, Li Y, Zhu S (2013). Association of survivin polymorphisms with tumor susceptibility: a meta-analysis. PLoS One.

